# Exploring the molecular mechanism of glycyrrhetinic acid in the treatment of gastric cancer based on network pharmacology and experimental validation

**DOI:** 10.18632/aging.204718

**Published:** 2023-05-11

**Authors:** Xia Li, Yuhua Du, Shicong Huang, Yi Yang, Doudou Lu, Junfei Zhang, Yan Chen, Lei Zhang, Yi Nan, Ling Yuan

**Affiliations:** 1 College of Pharmacy, Ningxia Medical University, Yinchuan 750004, Ningxia Hui Autonomous Region, China; 2 College of Basic Medicine, Ningxia Medical University, Yinchuan 750004, Ningxia Hui Autonomous Region, China; 3 Traditional Chinese Medicine College, Ningxia Medical University, Yinchuan 750004, Ningxia Hui Autonomous Region, China; 4 College of Clinical Medicine, Ningxia Medical University, Yinchuan 750004, Ningxia Hui Autonomous Region, China; 5 Key Laboratory of Hui Ethnic Medicine Modernization of Ministry of Education, Ningxia Medical University, Yinchuan 750004, Ningxia Hui Autonomous Region, China

**Keywords:** glycyrrhetinic acid, gastric cancer, network pharmacology, molecular docking, MAPK signaling pathway

## Abstract

There is a wide range of pharmacological effects for glycyrrhetinic acid (GRA). Previous studies have shown that GRA could inhibit the proliferation of tumor cells, showing a promising value in the treatment of gastric cancer (GC). Nonetheless, the precise mechanism of the effect of GRA on GC remains unclear. We explored cellular and molecular mechanisms of GRA based on network pharmacology and *in vitro* experimental validation. In this study, we predicted 156 potential therapeutic targets for GC with GRA from public databases. We then screened the hub targets using protein-protein interaction network (PPI) and conducted clinical correlation analysis. Gene Ontology (GO) enrichment and Kyoto Encyclopedia of Genes and Genomes (KEGG) pathway enrichment showed that GRA made anti-GC effects through multiple targets and pathways, particularly the MAPK signaling pathway. Next, molecular docking results revealed a potential interaction between GRA and MAPK3. In addition, qRT-PCR experiments revealed that 18β-GRA was able to suppress mRNA expression of *KRAS*, *ERK1* and *ERK2* in AGS cells. Western blotting results also revealed that 18β-GRA was able to suppress the expression of KRAS and p-ERK1/2 proteins in AGS cells. Additionally, immunofluorescence assays revealed that 18β-GRA inhibited p-ERK1/2 nuclear translocation in AGS cells. These results systematically reveal that 18β-GRA may have anti-tumor effects on GC by modulating the MAPK signaling pathway.

## INTRODUCTION

Gastric cancer (GC) is a familiar digestive tract malignancy, ranking fifth for incidence and third for mortality worldwide [1]. GC can be caused by environmental factors, diet, Helicobacter pylori infection, and individual factors [2]. In addition, the occurrence of GC has been linked to gender, family history, history of gastric disease, and occupation. The main treatments for GC are drug therapy, chemotherapy, and surgery, however, they all have poor therapeutic results [3]. In the early phase, GC has inconspicuous clinical symptoms, so once it is detected, it is generally at a moderate to advanced stage and accompanied by metastasis, which makes detection and treatment even more challenging with its high incidence and insidious features. Unfortunately, the prognosis for current treatments for GC is typically poor [4, 5]. Thus, it is pretty significant to find effective drugs which can cure GC.

**Figure 1:**
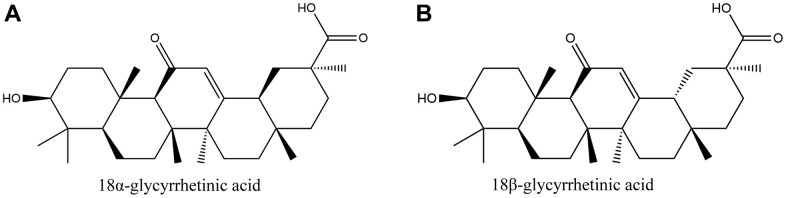
**The structure of glycyrrhetinic acid (GRA).** (**A**) 18α-glycyrrhetic acid (18α-GRA). (**B**) 18β-glycyrrhetic acid (18β-GRA).

Licorice is an extremely famous traditional Chinese medicine (TCM) that is commonly used in clinics. It is made from dried licorice roots and rhizomes and is available in the form of decoction pieces. Apart from its pharmacological value, licorice is also widely used in various foods for its nutritional and flavoring properties. Modern research has identified flavonoids, triterpenes, and polysaccharides as the main biologically active compounds in licorice [6, 7]. Glycyrrhetinic acid (GRA), an oleanane-type pentacyclic triterpenoid compound, is one of the major active components of licorice. GRA has multiple pharmacological effects, including anti-inflammatory, anti-tumor, antiviral, immunomodulatory, and similar [8–11]. GRA has two optical isomers, 18α-GRA and 18β-GRA. Their structures are shown below (Figure 1). 18β-GRA has a stronger antitumor effect than 18αGRA, and it has been found that 18β-GRA has a significant inhibitory effect in multiple types of tumors, such as breast cancer [12], hepatocellular cancer [13], lung cancer [14], ovarian cancer [15], and gastric cancer [16]. The main mechanisms of 18β-GRA can not only inhibit tumor cell proliferation, and promote tumor cell apoptosis, but also inhibit tumor cell invasion and migration. Of course, it also can inhibit tumor angiogenesis [17]. Previous studies have demonstrated that 18β-GRA can improve the inflammatory microenvironment by downregulating COX-2 and upregulating miR-149-3p to inhibit Wnt-1, thereby inhibiting the occurrence and progression of GC [18]. In addition, some studies have reported that 18β-GRA can inhibit the invasion and migration of GC cells through the ROS/PKC-α/ERK pathway [19]. Recently, it was found that 18β-GRA can regulate the apoptosis signaling pathway associated with MRPL35, and inhibit GC cells proliferation [20].

**Figure 2:**
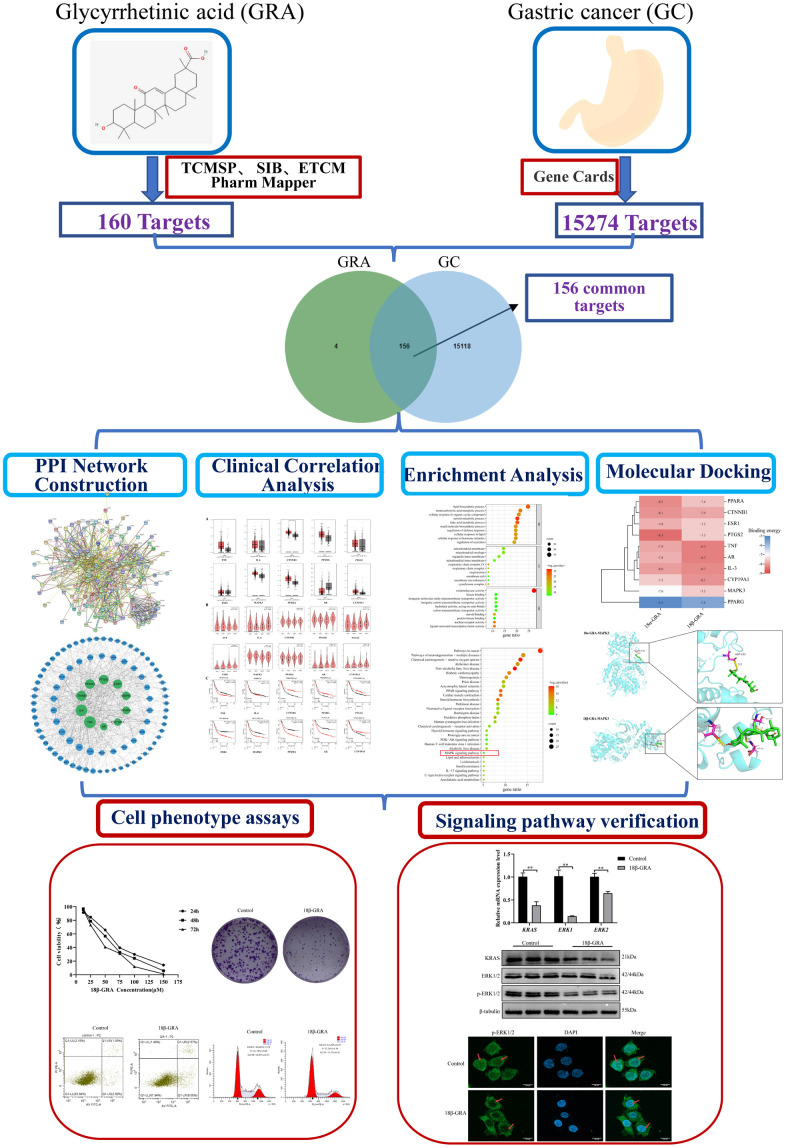
Workflow of the present study in a graphical manner.

Network pharmacology is an essential component of bioinformatics, which combines bioinformatics with systems medicine. Network pharmacology embodies the multi-component, multi-functional, and multi-faceted characteristics of TCM. Recently, people have used it to research TCM [21–23]. We predicted the potential mechanism for GRA in GC treatment through network pharmacology, designed experiments to test these predictions, and proposed different directions and ideas for future research. This study workflow is illustrated in Figure 2.

## RESULTS

### Common targets mining

**Figure 3:**
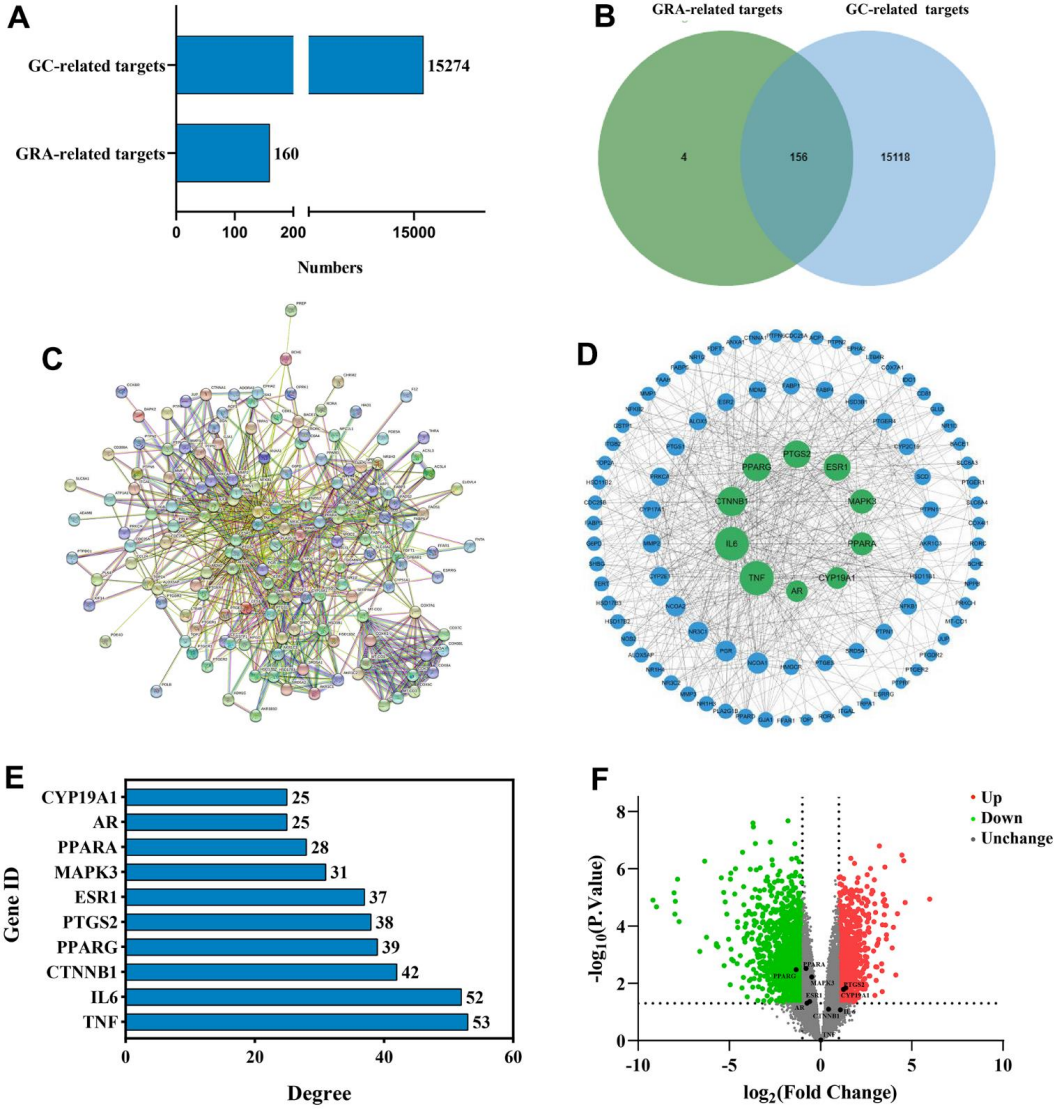
**Identification of common targets and analysis of PPI network.** (**A**) Potential targets of GRA-related and GC-related. (**B**) Venn diagram was applied to obtain the common targets between the GC targets and GRA targets. (**C**) PPI network of 156 common targets constructed with STRING. (**D**) The hub targets of PPI network. Larger node sizes indicate higher degree, green indicates higher degree, and blue indicates lower degree. (**E**) The PPI network’s 10 hub targets ranked by degree ≥ 25. (**F**) Volcano plot of differentially expressed genes in GC. Red represents upregulated genes and green represents downregulated genes.

We used network pharmacology to predict GRA’s potential mechanism in the GC therapy and obtained 160 GRA and 15274 GC relevant targets with the target prediction website (Figure 3A). Afterwards, we made a Venn diagram for the GRA and GC targets, and screened 156 common targets (Figure 3B).

### Constructing the PPI network and analysing hub targets

To further explore the relationship between 156 common targets, we analyzed the common targets through the STRING website (Figure 3C) and obtained the PPI network. We analyzed the PPI network using Cytoscape 3.8.2 (Figure 3D). On the basis of degree value analysis, the top 10 hub targets in the PPI network are TNF (degree = 53), IL-6 (degree = 52), CTNNB1 (degree = 42), PPARG (degree = 39), PTGS2 (degree = 38), ESR1 (degree = 37), MAPK3 (degree = 31), PPARA (degree = 28), AR (degree = 25), and CYP19A1 (degree = 25) (Figure 3E). The common target’s degree value were shown in Supplementary Table 1. DEGs were analyzed by the GEO database. We used |log2(fold change)| > 1 and *p* < 0.05 in the volcano diagram, with red representing upregulated genes and green representing downregulated genes. *PPARG* (*p* = 0.00333) obviously downregulated, meanwhile *PTGS2* (*p* = 0.0144) and *CYP19A1* (*p* = 0.00664) obviously upregulated (Figure 3F).

**Figure 4:**
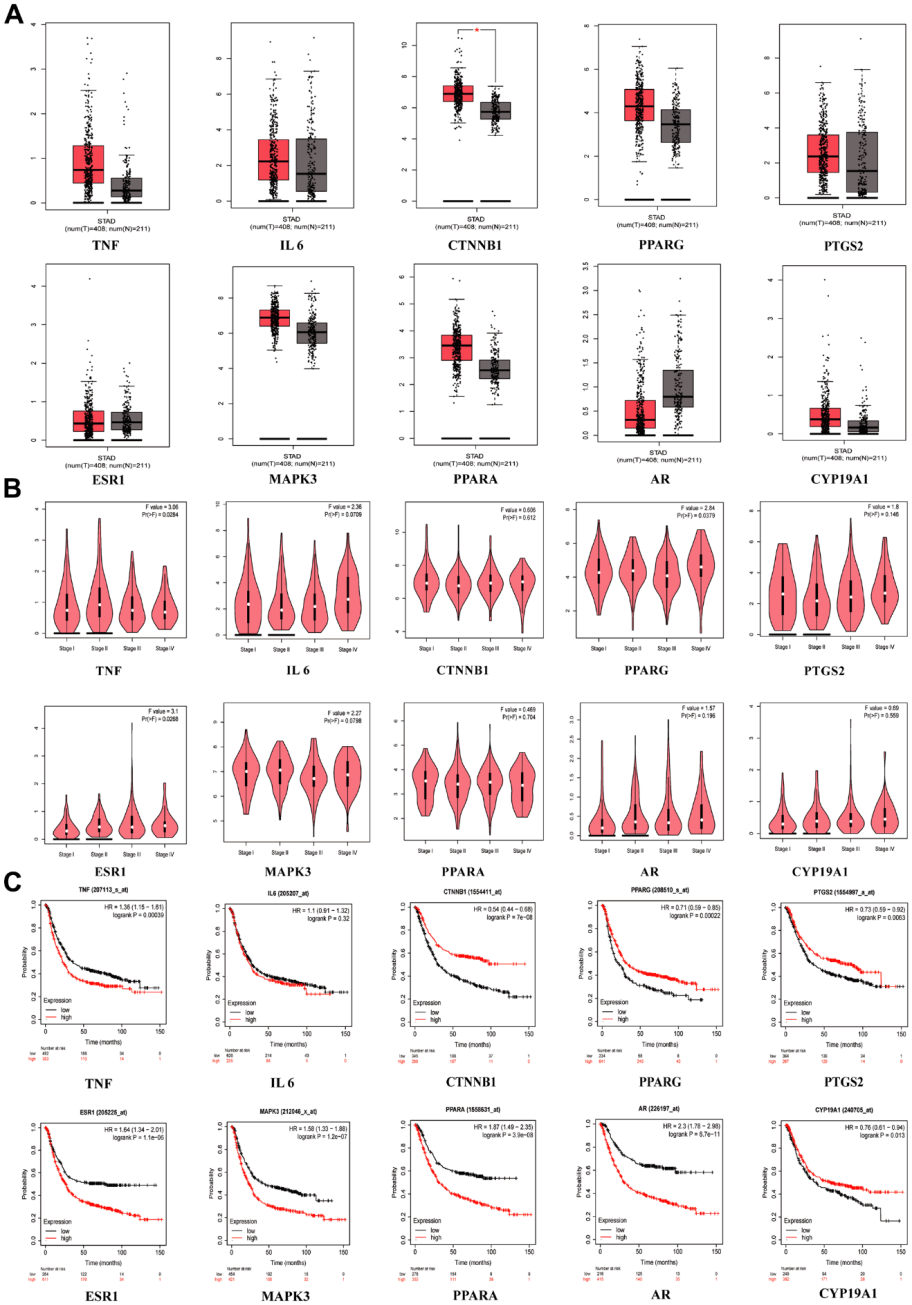
**Clinical correlation analysis of hub targets.** (**A**) mRNA expression level of hub targets between GC tissues and normal gastric tissues, *p <0.05. (**B**) The significance of hub target in staging of GC, *p* <0.05 is considered to be statistically significant. (**C**) Survival analysis of hub targets, Log-rank *p* <0.05 is considered to be statistically significant.

When we compared the normal group to the GC group in GEPIA, we discovered that *CTNNB1* mRNA expression was upregulated (Figure 4A). *TNF* (*p* = 0.0284), *PPARG* (*p* = 0.0379) and *ESR1* (*p* = 0.0268) were significant in tumor staging in GC patients (Figure 4B). We found that patients who had higher expression of *TNF* (*p* = 0.00039), *ESR1* (*p* = 1.1E-06), *MAPK3* (*p* =1.2E-07), *PPARA* (*p* = 3.9E-06) and AR(*p* =6.7E-11) had worse survival than patients who had lower expression, while the opposite was true for *CTNNB1* (*p* = 7E-08), *PTGS2* (*p* = 0.0083), *PPRAG* (*p* = 0.00022) and *CYP19A1* (*p* = 0.013), in the Kaplan-Meier plotter database (Figure 4C). When the *p* < 0.001 was reached, we considered the gene to be a prognostic marker for GC (Criteria for prognostic markers were reference to The Human Protein Atlas website). Based on the *p* value, *TNF*, *CTNNB1*, *PPARG*, *ESR1*, *MAPK3*, *PPARA* and *AR* can be used as prognostic markers of GC.

### GO and KEGG enrichment analysis

**Figure 5:**
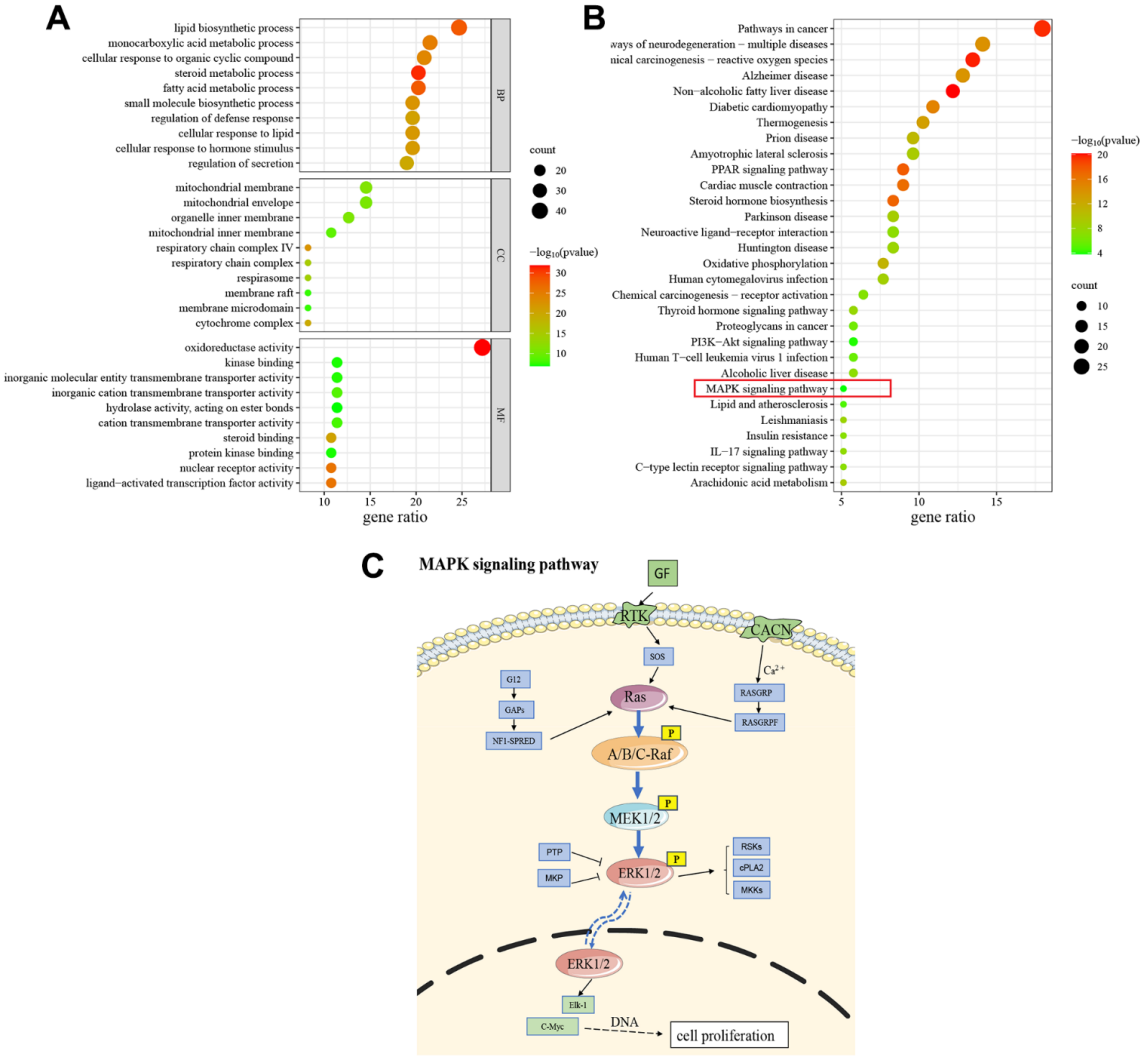
**GO and KEGG enrichment analysis.** (**A**) The results of GO enrichment analysis (Top 10). (**B**) The results of KEGG pathway enrichment analysis (Top 30). (**C**) Schematic drawing of the MAPK signaling pathway.

We performed it to further explore the potential mechanisms of GRA treatment of GC, The top 10 enrichment terms of biological processes (BP), cellular components (CC), and molecular function (MF) were presented in a bubble diagram (Figure 5A). The results of GO enrichment analysis suggested that common targets were mainly related to BP and metabolic process, such as lipid biosynthetic process, monocarboxylic acid metabolic process, steroid metabolic process, fatty acid metabolic process, and small molecule biosynthetic process. The MF mainly involved enzyme activity, transmembrane transporter activity, and receptor activity. Additionally, the CC were primarily associated with protein complexes and mitochondria. We also performed KEGG pathway enrichment analysis for common targets and the results showed that GRA modulates GC progression through multiple pathways associated with survival, differentiation, division and apoptosis (Figure 5B). Among them, MAPK signaling pathway is often activated in tumors, and its related proteins’ abnormal expression and tumors’ occurrence go hand in hand (Figure 5C).

### Molecular docking

**Figure 6:**
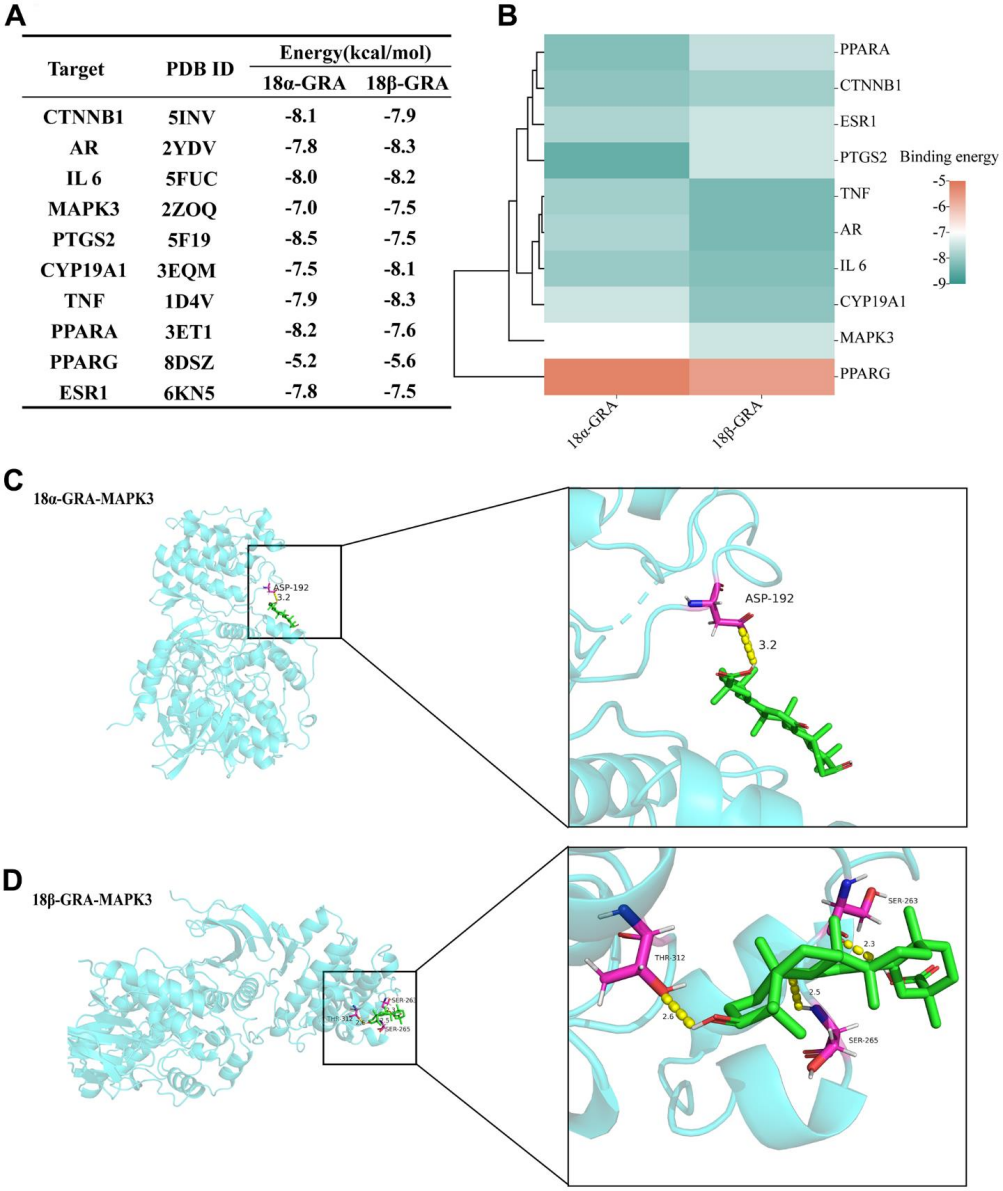
**The results of molecular docking.** (**A**) The results of molecular docking binding energy. (**B**) Heat map of molecular docking binding energy. (**C**) 18α-GRA and MAPK3 molecular docking visualization. (**D**) 18β-GRA and MAPK3 molecular docking visualization.

Molecular docking verifies the binding energy of GRA and hub targets. The results of 18α-GRA and 18β-GRA docking with hub targets (Figure 6A). A binding energy < − 5.0 kcal/mol indicates excellent binding capability. The results showed that GRA can be combined with all the hub targets (Figure 6B). 18α-GRA and MAPK3 binding energy was -7.0 kcal/mol (Figure 6C), and 18β-GRA to MAPK3 was -7.5 kcal/mol (Figure 6D).

### 18β-GRA inhibited the GC cells proliferation

**Figure 7:**
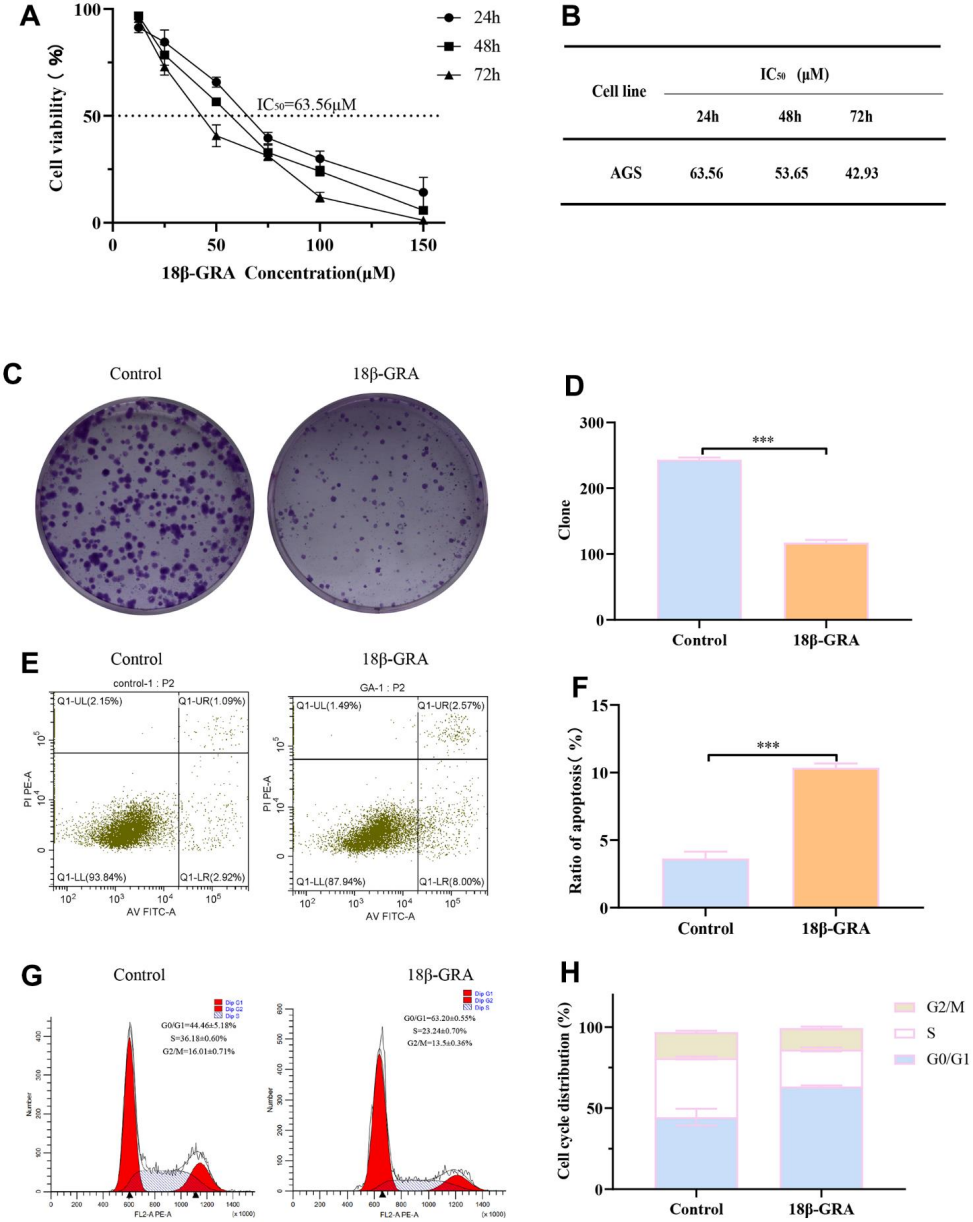
**The effect of 18β-GRA on the phenotype of AGS cells.** (**A**) The effect of 18β-GRA on AGS cells viability. (**B**) IC50 values of 18β-GRA interfered with AGS cells after 24 h, 48 h, and 72 h. (**C**, **D**) The results of colony formation and statistical chart. (**E**, **F**) The results of cell apoptosis and statistical chart. (**G**, **H**) The results of cell cycle and statistical chart. All the values are expressed as mean ± SD. Compared with the control group, ***p < 0.001.

Consequently, we designed experiments to validate our prediction. 18β-GRA’s effect on AGS cells viability was demonstrated by the CCK-8 method. We treated AGS cells with different concentrations of 18β-GRA for 24 h, 48 h, and 72 h for cell viability assay. The results demonstrated that 18β-GRA could significantly decrease AGS cells viability in a dose-dependent manner (Figure 7A). The IC_50_ of 18β-GRA intervention on AGS cells is shown in Figure 7B. The IC_50_ of AGS cells treated with 18β-GRA for 24 h was 63.56 μM, so 63.56 μM intervention for 24 h was the dose administered in our subsequent experiments. As shown in Figure 7C, 7D, 18β-GRA effect on AGS cells colony formation ability was observed. The results showed that the cell colony formation ability of 18β-GRA intervention group was obviously lower (*p* < 0.001) compared with the control group.

### 18β-GRA promoted GC cells apoptosis and arrested cell cycle

To further explore the ability of 18β-GRA to inhibit AGS cells proliferation, we analyzed the cells cycle in AGS and apoptosis with 18β-GRA intervention in AGS cells. Flow cytometry function was to confirm the effect of 18β-GRA on AGS cells apoptosis. The results illustrated that 18β-GRA made AGS cells apoptotic rate increase from 3.64 ± 0.50% to 10.67 ± 0.31% (*p* < 0.001) (Figure 7E, 7F). 18β-GRA effect on cell cycle distribution was detected. The results suggested that the G0/G1 proportion increased in the 18β-GRA intervention group (Figure 7G, 7H). The G0/G1 proportion was 63.20 ± 0.55%, which was higher than the control group’s 44.64 ± 5.18% (*p* < 0.001). The above results indicated that 18β-GRA can effectively promote AGS cells apoptosis, arrest the AGS cell cycle in the G0/G1 phase, and thus have certain effects on AGS cells proliferation.

### 18β-GRA inhibited the related targets expression of MAPK signaling pathway

**Figure 8:**
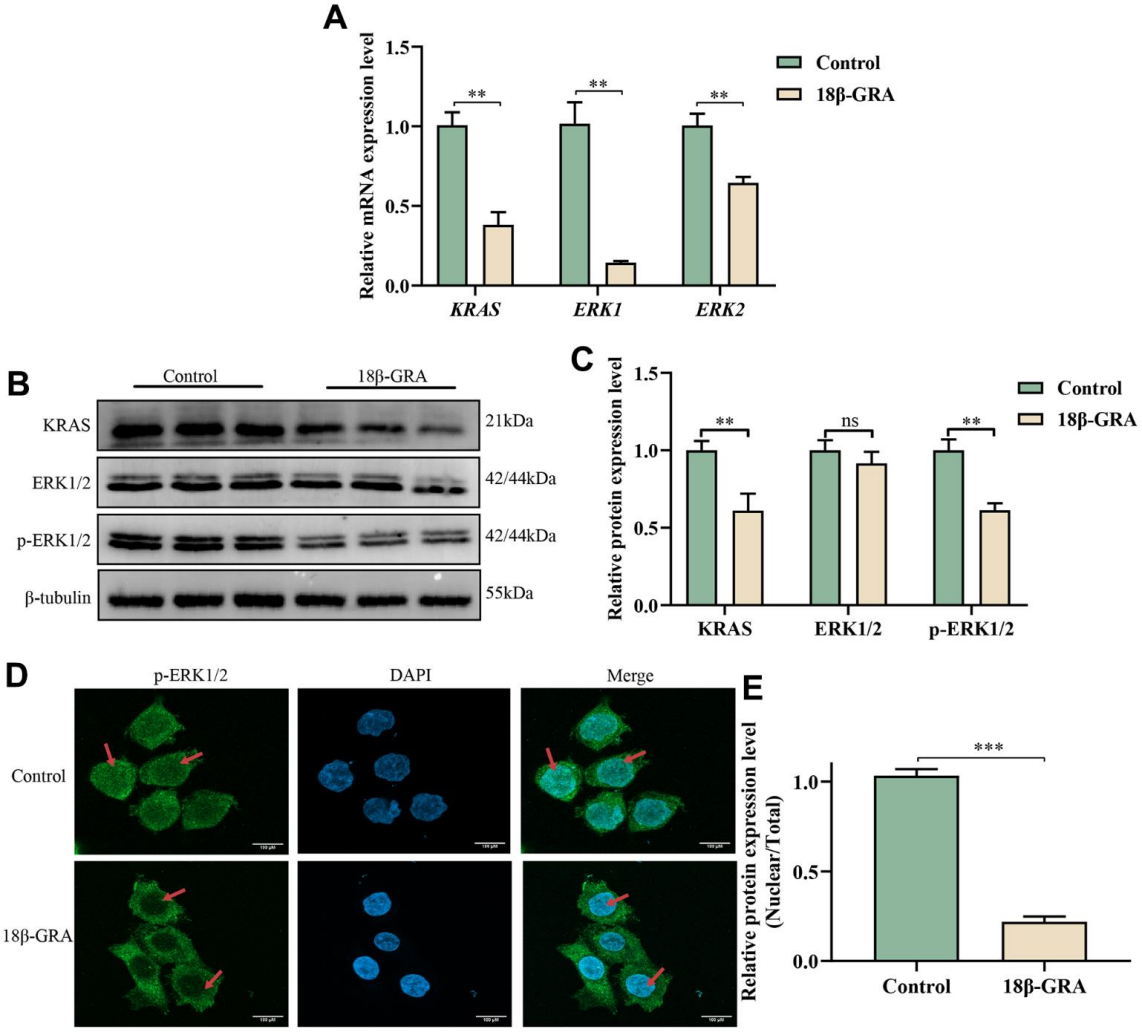
**The effect of 18β-GRA on MAPK pathways in AGS cells.** (**A**) qRT-PCR detection of *KRAS*, *ERK1*, and *ERK2* expression levels. (**B**) Western blotting analysis revealed the presence of KRAS, ERK1/2, p-ERK1/2 protein expression levels. (**C**) The results quantify protein levels of KRAS, ERK1/2 and p-ERK1/2 used Image J software. (**D**, **E**) The effects of 18β-GRA on nuclear transfer of p-ERK1/2 in AGS cells and statistical chart. All the values are expressed as mean ± SD. Compared with the control group, **p < 0.01, ***p < 0.001.

To identify the expression of hub targets in the MAPK signaling pathway, we used western blotting, qRT-PCR, and immunofluorescence techniques. The results of the qRT-PCR experiment demonstrated that the mRNA expression levels of *KRAS* (*p* < 0.01), *ERK1* (*p* < 0.01) and *ERK2* (*p* < 0.01) obviously decreased in 18β-GRA intervention group (Figure 8A). According to the results of western blotting, KRAS (*p* < 0.01) and p-ERK1/2 (*p* < 0.01) decreased in the 18β-GRA intervention group, and ERK1/2 did not alter significantly (*p* > 0.05) (Figure 8B, 8C).

An immunofluorescence assay was employed to find the nuclear translocation of p-ERK1/2 brought on by 18β-GRA intervention. The results demonstrated that the p-ERK1/2 expression in the cell nucleus decreased in the 18β-GRA intervention group (*p* < 0.001) (Figure 8D, 8E).

## DISCUSSION

For thousands of years, the Chinese have used natural herbs in clinical settings. Nowadays, it has been attracting increasing attention due to its wide range of pharmacological effects. Numerous studies have shown that some herbal extracts and monomeric components have an essential anticancer role [24]. TCM has multiple targets, pathways, and mechanisms of action [25], but it is challenging to clarify them. Recently, the combination of bioinformatics analysis and pharmacology has been complemented by network pharmacology. People use it to systematically elucidate the mechanisms of TCM.

This study utilized public databases to predict the relationship between GRA and GC, and analyzed the PPI network to identify hub targets. The key target MAPK3 in the MAPK signaling pathway ranks higher in the PPI network. Further analysis showed that MAPK3 was closely related to GC staging and prognosis in clinical correlation analysis. GO functional enrichment analysis identified common targets of GRA in the treatment of GC involved transmembrane transporters and nuclear receptor proteins, and KEGG enrichment results predicted that the MAPK signaling pathway may be a potential pathway for GRA in GC therapy. In MAPK signaling pathway, KRAS is located on the cell membrane, and p-ERK1/2 can participate in signal transduction across the nuclear membrane. Furthermore, molecular docking predicted that GRA could strongly bind with MAPK3, one of the hub targets. To further verify the prediction, we conducted *in vitro* experiments to investigate the therapeutic potential of 18β-GRA on GC cells. The results showed that 18β-GRA could obviously inhibit GC cells proliferation, promote cell apoptosis, and arrest cell cycle.

It is well known that cell apoptosis plays an important role in various biological processes related to tumorigenesis [26]. The maintenance of cellular homeostasis relies on the balance between pro-apoptotic and anti-apoptotic signals [27], and the disruption of apoptotic regulatory pathways is a significant contributor to carcinogenesis [28]. Insufficient apoptosis resulting from a deficiency of appropriate pro-apoptotic signaling pathways or increase activity of anti-apoptotic factors can lead to continued proliferation of cancer cells. The processes of cell proliferation and cell cycle are inextricably linked [29]. Cell cycle progression involves the activation of various complexes that prevent cell from entering a new phase until the previous phase is successfully completed. Our results indicated that 18β-GRA promoted GC cells apoptosis and arrested cell cycle in the G0/G1 phase, thereby inhibiting their proliferation.

Tumor proliferation and growth relied on the activation and regulation of multiple signaling pathways. The MAPK signaling pathway is frequently activated in tumors and plays a crucial role in regulating cell growth, development, survival, differentiation, division and apoptosis [30]. ERK1 and ERK2, mitogen-activated protein kinases (MAPKs), are cytoplasmic kinases that are activated in response to various stimuli and are key elements of signaling from the cell surface to the interior of the cell [31]. They are also essential components in signal transduction from the surface to the interior of the cell. The MAPK signaling pathway plays an essential role in regulating cell growth, development and division. The pathway is composed of MAP3K, MAP2K, and MAPK, and follows a three-stage enzymatic reaction. Ras is a guanosine triphosphatase (GTPase) located in the cell membrane, which binds GTP proteins and activates the phosphorylation cascade to deliver cellular signals. Ras has three isoforms, namely K-Ras, H-Ras, and N-Ras, which are expressed widely [32]. The Ras-Raf-MEK-ERK signaling pathway consists of Ras acting as an upstream activator protein, MEK1/2 as a MAP2K, and ERK1/2 as a MAPK [33, 34]. Once activated, p-ERK1/2 can be transported to the nucleus, where it binds to transcription factors, ultimately affecting the expression of cell-related genes. Previous studies have shown that ERK1/2 is abundantly expressed in various human tumors, including GC, hepatocellular carcinoma, glioblastoma multiforme, breast cancer, and lung cancer [35–39]. In this study, the results of western blotting and qRT-PCR demonstrated that treatment with 18β-GRA in GC cells led to significant suppression of KRAS and p-ERK1/2 expression in the MAPK signaling pathway. Additionally, immunofluorescence experiments showed that 18β-GRA could affect the nuclear translocation of p-ERK1/2 and block its entry into the nucleus in GC cells. These findings provide evidence that 18β-GRA may have anti-GC properties by suppressing the expression of KRAS and p-ERK/2 in the MAPK signaling pathway, as well as blocking p-ERK1/2 from entering the nucleus and binding to related transcription factors in the nucleus.

In summary, this study showed that 18β-GRA can reduce GC cells clone formation ability, promote cell apoptosis and arrest cell cycle in the G0/G1 phase by suppressing the MAPK signaling pathway and thus inhibiting the proliferation of GC cells, which could provide a scientific basis for the related research of 18β-GRA in the treatment of GC. Nevertheless, our research is limited to *in vitro* cell experiments, and more experiments are needed to support our future research. Therefore, we will continue to explore the link between 18β-GRA and GC in the future with the following study. First, the therapeutic effect of 18β-GRA on GC is investigated by *in vivo* animal experiments. Second, to explore the effect of 18β-GRA in reducing chemotherapeutic drug sensitivity in combination with chemotherapy drugs. Third, the molecular mechanism of 18β-GRA in GC treatment through gene silencing, co-IP, EMSA, and other methods should be further investigated.

## CONCLUSIONS

In this study, we investigated the molecular mechanism of 18β-GRA in the treatment of GC through network pharmacology and experimental verification. The results showed that 18β-GRA could inhibit the proliferation of GC cells by suppressing the MAPK signaling pathway, induce apoptosis, arrest cell cycle, and reduce colony forming ability. Our results confirm the reliability of network pharmacology analysis and provide a strong scientific basis for further research.

## MATERIALS AND METHODS

### Acquisition of GRA-related targets

We obtained the 2D or 3D structure of GRA by PubChem (https://pubchem.ncbi.nlm.nih.gov/). The TCMSP database (https://tcmsp-e.com/), the PharmMapper database (https://www.lilab-ecust.cn/pharmmapper/), the ETCM database (http://www.tcmip.cn/ETCM/index.php/Home/) and the SIB database (http://www.swisstargetprediction.ch/) were used to predict GRA targets. The search results were combined and deduplicated to obtain GRA-related targets.

### Acquisition of GC-related targets

We searched the keywords “gastric cancer,” “stomach neoplasm,” “stomach cancer” and “gastric carcinoma,” from the GeneCards database (https://www.genecards.org/). Then, we combined and deduplicated the search results to obtain GC-related targets.

### Protein-protein interaction (PPI) network analysis

We used the Venny 2.1.0 platform (https://bioinfogp.cnb.csic.es/tools/venny/index.html) to obtain the common targets of GRA and GC, and make a Venn diagram. We entered the common targets into the STRING database (https://string-db.org/), selected Homo sapiens, and set the confidence range to “scoring value >0.7”. Next, we downloaded the TSV file and uploaded it to the Cytoscape 3.8.2 to make the PPI network and filter hub targets based on degree ranking.

### Clinical correlation analysis of hub targets

We downloaded GC patients’ differentially expressed genes (DEGs) from the GEO database (https://www.ncbi.nlm.nih.gov/geo/), series: GES79973, then adjusted for *p* < 0.05 and | log 2 (fold change) | > 1. We used the GEPIA (http://GEPIA.cancer-pku.cn/) to obtain the expression levels of hub target gene in GC and normal tissues, and analyzed the differences and changes in expression level at different stages. We used the Kaplan-Meier plotter database (http://kmplot.com/analysis/) to analyze the hub target genes’ influence on the prognosis of GC patients.

### Enrichment analysis

We used the Metscape database (https://metascape.org/gp/index.html#/main/step1) to analyze Gene Ontology (GO) and Kyoto Encyclopedia of Genes and Genomes (KEGG) of common targets. Sort by the number of enrichments. We visualized the results as a bubble diagram.

### Molecular docking

We obtained the protein structures of the hub targets from the PDB database (https://www.rcsb.org/) and used chem3D software to convert 2D structure to a 3D structure of GRA. We used the Auto Dock Tools to modify the protein [24], perform molecular docking of the receptor and the ligand, and evaluate the binding energy. We visualized the molecular docking results by Pymol.

### Cell culture

Human GC cell line AGS cells (Cat. No. CL-0022, Procell, China) were cultured in DMEM/F12 medium which contain 10% fetal bovine serum (FBS, Cat. No. SH30256, Gibco, USA) in a humidity incubator with 5% CO_2_ at 37° C.

### Cell viability assay

We inoculated AGS cells in 96-well plates at 5×10^4^ cells/ml and incubated for 24 h. Next, we added 18β-GRA (Cat. No. G10105-10G, Sigma, USA) at concentrations of 0-150 μM incubation was continued for 24 h, 48 h and 72 h. Then, we added 10 μl CCK-8 (Cat. No. KGRA317, KeyGEN, China) to each well and incubated for 2 h at 37° C. Finally, we detected optical density (OD) at 450 nm. All experimental groups repeated 4 wells.

### Colony formation assay

We inoculated AGS cells in 6-well plates with 500 cells per well and incubated for 24 h, intervened with 18β-GRA and incubated for approximately 14 days. We used 4% paraformaldehyde to fixate the cells, crystal violet to stain, and distilled water to wash. Finally, the cell clones were photographed and statistical analysis based on clone sizes (Diameter > 1 mm). All experimental groups repeated 3 samples.

### Cell apoptosis and cell cycle assay

We inoculated AGS cells in culture flask and incubated for 24 h, intervened with 18β-GRA. Next, we collected the cells and stained with an apoptosis detection kit (Cat. No. KGRA107, KeyGEN, China), then detected by flow cytometry. We collected the cells from each group, washed with PBS, and fixed overnight at 4° C with 70% ethanol. We stained the cells with a cell cycle kit (Cat. No. KGRA512, KeyGEN, China), and detected by flow cytometry. All experimental groups repeated 3 samples.

### Quantitative real-time polymerase chain reaction (qRT-PCR)

We extracted the total RNA from AGS cells with Trizol (Cat. No. DP419, Tiagen Biochemical Technology, China). Subsequently, we synthesized cDNA based on the instructions of the PrimeScriptTM RT kit (Cat. No. RR047A, TaKaRa, Japan). We performed qRT-PCR with the SYBR Green kit (Cat. No. FP205, Tiagen Biochemical Technology, China). The expression levels of Kirsten rat sarcoma viral oncogene (*KRAS*), extracellular-regulated protein kinase 1 *(ERK1)*, and extracellular-regulated protein kinase 2 (*ERK2*) were quantified. The primer sequences are as follows: *KRAS*: forward: 5’-TGTGGACGAATATGATCCAACA-3’, reverse: 5’- GCAAATACACAAAGAAAGCCCT-3’; *ERK1*: forward: 5’-ATGTCATCGGCATCCGAGAC-3’, reverse: 5’- GGATCTGGTAGAGGAAGTAGCA -3’; *ERK2*: forward: 5’- TACACCAACCTCTCGTACATCG -3’, reverse: 5’- ATGTCTGAAGCGCAGTAAGATT -3’; *GAPDH*: forward: 5’- CACCCACTCCTCCACCTTTGA -3’, reverse: 5’- TCTCTCTTCCTCTTGTGCTCTCTTGC -3’. *GAPDH* was used as an internal reference gene. All experimental groups repeated 3 samples.

### Western blotting

Western blotting detected changes in protein expression. 18β-GRA intervened in AGS cells for 24 h. We extracted the total protein by RIPA lysis buffer (Cat. No. PC102, Epizyme Biotech, China), assessed the protein content by the BCA method. Next, we separated the total protein by SDS-PAGE, transferred onto PVDF membranes (Cat. No. ISEQ00010, Millipore, USA), and sealed the membrane with 5% BSA for 1 h. After that, we incubated the membranes in the corresponding primary antibody overnight at 4° C. The corresponding primary antibodies included anti-KRAS (Cat. No. ab275876, Abcam, 1:1000), anti-ERK1/2 (Cat. No. 9102, CST, 1:2000), anti-p-ERK1/2 (Cat. No. 4370, CST, 1:2000), and anti-β-tubulin (Cat. No. 2146, CST, 1:5000). Moreover, we used TBST to wash the membranes 3 times and incubated them with anti-mouse/rabbit IgG antibodies (Cat. No. S0001 / S0002, Affinity, 1:5000) for 1 h. Finally, we detected the proteins by ECL and measured band intensities by ImageJ. All experimental groups repeated 3 samples.

### Immunofluorescence

We treated AGS cells with 18β-GRA and fixated the cells with 4% paraformaldehyde. Afterwards, we used PBS to wash the cells three times, 0.5% Triton X-100 solution to treat for 15 min, and 5% BSA to seal for 1 h. After that, we used the primary antibody (p-ERK1/2, 1: 500) to incubate the cells overnight at 4° C. The next day, we used PBS to wash the cells again, added the fluorescent secondary antibody (Cat. No. 111-545-144, Jackson ImmunoResearch Inc, USA), and then incubated for 1 h. Subsequently, we stained the nucleus with DAPI for 5 min. We used confocal scanning microscopy (LSM 900, Zeiss, Germany) to take photos. All experimental groups repeated 3 samples.

### Statistical analysis

All data were analyzed using GraphPad Prism 7 software and presented as the mean ± SD of at least three independent samples. Using t-tests analyzed the statistical differences, *p* < 0.05 indicated a significant difference.

## Supplementary Material

Supplementary Table 1
