# Supplementary material for: Exploring the molecular mechanism of glycyrrhetinic acid in the treatment of gastric cancer based on network pharmacology and experimental validation
Source: Aging (Albany NY). 2023 May 11;15(9):3839–56. doi: 10.18632/aging.204718 (PMC10449304; doi:10.18632/aging.204718)
Supplement: Supplementary Table 1 [file aging-15-204718-s001.pdf]

SUPPLEMENTARY TABLE

Supplementary Table 1. The common target's degree value.

Number	Targets	Degree
1	TNF	53
2	IL6	52
3	CTNNB1	42
4	PPARG	39
5	PTGS2	38
6	ESR1	37
7	MAPK3	31
8	PPARA	28
9	AR	25
10	CYP19A1	25
11	NCOA1	24
12	PGR	23
13	NR3C1	22
14	NCOA2	21
15	CYP2E1	19
16	CYP17A1	18
17	MMP2	18
18	PTGS1	17
19	PRKCA	17
20	ESR2	16
21	ALOX5	16
22	MDM2	16
23	FABP4	15
24	FABP1	15
25	HSD3B1	15
26	NFKB1	14
27	SCD	14
28	PTPN11	14
29	AKR1C3	14
30	HSD11B1	14
31	PTGER4	14
32	CYP2C19	14
33	PTPN1	13
34	SRD5A1	13
35	PTGES	13
36	GJA1	12
37	PPARD	12
38	HMGCR	12
39	MMP3	11
40	NR1H4	11
41	NR1H3	11
42	PLA2G1B	11
43	NR3C2	11
44	TERT	10
45	NOS2	10
46	HSD17B2	10
47	HSD17B3	10
48	ALOX5AP	10
49	SHBG	10
50	FABP3	9
51	G6PD	9
52	CDC25B	9
53	MMP1	8
54	HSD11B2	8
55	GSTP1	8
56	ITGB2	8
57	NFKB2	8
58	TOP2A	8
59	CTNNA1	7
60	FABP5	7
61	FDFT1	7

62	ANXA1	7
63	FAAH	7
64	NR1I2	7
65	GLUL	6
66	NR1I3	6
67	PTPN6	6
68	ACP1	6
69	PTPN2	6
70	EPHA2	6
71	LTB4R	6
72	IDO1	6
73	CD81	6
74	BACE1	6
75	CDC25A	6
76	COX7A1	6
77	RORC	5
78	PTGER1	5
79	SLC6A3	5
80	SLC6A4	5
81	COX4I1	5
82	MT-CO1	4
83	JUP	4
84	NPPB	4
85	PTGDR2	4
86	PTGER2	4
87	PRKCH	4
88	BCHE	4
89	RORA	3
90	PTPRF	3
91	ESRRG	3
92	TRPA1	3
93	ITGAL	3
94	TOP1	3
95	FFAR1	2
